# 2-{(*E*)-[1-(2-Hydroxy­ethyl)-3,3-dimethyl-3*H*-indol-1-ium-2-yl]vin­yl}-6-hydroxy­meth­yl-4-nitro­phenolate dihydrate

**DOI:** 10.1107/S1600536809027238

**Published:** 2009-07-18

**Authors:** Mark A. Rodriguez, Greg O’Bryan, William J. Andrzejewski, James R. McElhanon

**Affiliations:** a PO Box 5800, MS 1411, Sandia National Laboratories, Albuquerque, NM 87185, USA; b PO Box 969, MS 9403, Sandia National Laboratories, Livermore, CA 94551, USA; c PO Box 5800, MS 01455, Sandia National Laboratories, Albuquerque, NM 87185, USA; d PO Box 5800, MS 0888, Sandia National Laboratories, Albuquerque, NM 87185, USA

## Abstract

The title merocyanine-type mol­ecule, C_21_H_22_N_2_O_5_·2H_2_O, crystallizes in a zwitterionic form and has an *E* configuration at the styryl C=C bond. The styryl part of the mol­ecule and the indolium ring are slightly twisted and form a dihedral angle of 13.4 (1)°. The 1.274 (3) Å C—O bond length in the phenolate fragment is the longest among similar mol­ecules. Hydrogen bonds between solvent water mol­ecules, two hydroxyl groups and the phenolate O atom dictate the packing arrangement of mol­ecules in the crystal and join the mol­ecules into a two-dimensional polymeric network which propagates parallel to (001). Four water mol­ecules and four hydr­oxy groups form a centrosymmetric homodromic cyclic motif of O—H⋯O hydrogen bonds. Another cyclic centrosymmetric motif is generated by four water mol­ecules and two phenolate O atoms.

## Related literature

This structure is similar to the perviously reported *trans*-MEH compound, see: Raymo *et al.* (2003 ▶). For similar structures, see also: Aldoshin & Atovmyan (1985 ▶), Hobley *et al.* (1999 ▶), Zou *et al.* (2003 ▶). For the synthetic procedure, see: Raymo & Giordani (2001 ▶). 
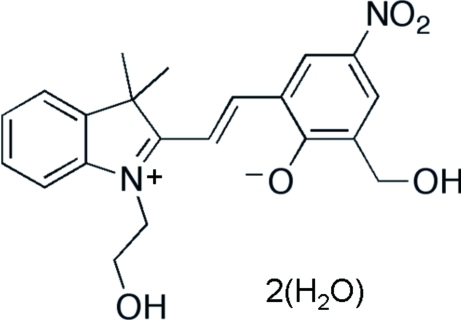


## Experimental

### 

#### Crystal data

C_21_H_22_N_2_O_5_·2H_2_O
*M* *_r_* = 418.44
Triclinic, 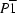

*a* = 7.377 (2) Å
*b* = 8.868 (2) Å
*c* = 16.817 (5) Å
α = 94.603 (5)°
β = 101.639 (6)°
γ = 102.140 (7)°
*V* = 1044.8 (5) Å^3^
*Z* = 2
Mo *K*α radiation
μ = 0.10 mm^−1^
*T* = 183 K
0.10 × 0.10 × 0.10 mm

#### Data collection

Bruker APEX CCD area-detector diffractometer
Absorption correction: multi-scan (*SADABS*; Sheldrick, 1999 ▶) *T*_min_ = 0.981, *T*_max_ = 0.990
7525 measured reflections
3651 independent reflections
2472 reflections with *I* > 2σ(*I*)
*R*_int_ = 0.039

#### Refinement

*R*[*F* ^2^ > 2σ(*F* ^2^)] = 0.054
*wR*(*F* ^2^) = 0.134
*S* = 1.02
3651 reflections
271 parameters
H-atom parameters constrained
Δρ_max_ = 0.23 e Å^−3^
Δρ_min_ = −0.20 e Å^−3^

### 

Data collection: *SMART* (Bruker, 1998 ▶); cell refinement: *SAINT-Plus* (Bruker, 2001 ▶); data reduction: *SAINT-Plus*; program(s) used to solve structure: *SHELXTL* (Sheldrick, 2008 ▶); program(s) used to refine structure: *XSHELL* (Bruker, 2000 ▶); molecular graphics: *XSHELL* and *Mercury* (Macrae *et al.*, 2008 ▶); software used to prepare material for publication: *SHELXTL*.

## Supplementary Material

Crystal structure: contains datablocks I, global. DOI: 10.1107/S1600536809027238/gk2221sup1.cif

Structure factors: contains datablocks I. DOI: 10.1107/S1600536809027238/gk2221Isup2.hkl

Additional supplementary materials:  crystallographic information; 3D view; checkCIF report

## Figures and Tables

**Table 1:** Hydrogen-bond geometry (Å, °)

*D*—H⋯*A*	*D*—H	H⋯*A*	*D*⋯*A*	*D*—H⋯*A*
O20—H20*B*⋯O10^i^	0.96	1.80	2.739 (4)	166
O10—H10*B*⋯O2^ii^	0.95	1.81	2.750 (3)	172
O20—H20*A*⋯O2	0.95	1.78	2.714 (3)	167
O10—H10*A*⋯O1^ii^	0.95	1.87	2.811 (3)	165
O5—H5⋯O20^iii^	0.84	1.80	2.633 (3)	175
O1—H1⋯O5^iv^	0.84	1.90	2.734 (3)	176

Symmetry codes: (i) 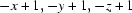; (ii) 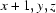; (iii) 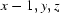; (iv) 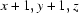.

**Fig. 1.**
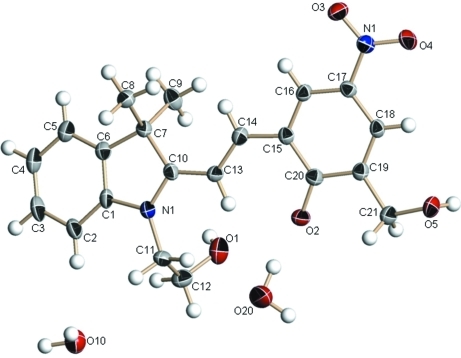
The molecular structure of the title compound, with labels and 50% probability displacement ellipsoids for non-H atoms.

**Fig. 2.**
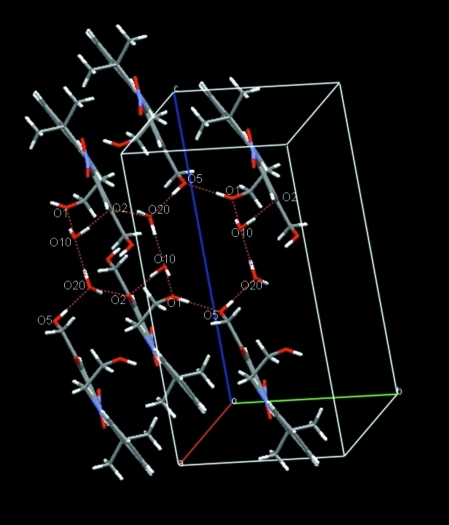
Packing diagram for the title compound showing solvent water interactions. See text for details.

## supplementary crystallographic information

### Comment

Figure 1 shows an atomic displacement ellipsoid plot of the title compound. The zwitterionic molecule is nearly planar, with a 13.4 (1)*^o^* diheral angle tilt between the plane generated from the phenolate portions of the molecule as compared to the plane associated with the indole ring portion of the molecule. Thermal ellipsoids for most of the atoms are well defined. Only the O20 oxygen atom associated with one of two solvent water molecules shows some enlargement, and such enlargement is not unexpected. The title compound is similar to another merocyanine molecule (*trans*-MEH) as documented by Raymo & Giordani (2001) and Raymo *et al.* (2003), with the difference being that the title compound possesses an additional methanol group on the phenolate portion of the molecule. A review of similar structures which contain terminal alkoxy ligands (C—O^-^) shows C—O bond lengths in the range of 1.228 to 1.260 Angstroms; see Aldoshin & Atovmyan (1985), Hobley, *et al.* (1999), and Zou, *et al.* (2003). The C—O^-^ bond for the title compound falls outside this range at 1.274 (3) Angstroms. This elongation is likely a result of H-bonding interactions as discussed below. Figure 2 shows the packing arrangement and intermolecular interactions for the title compound. One can see the nearly planar nature of the molecule from this perspective. There are two cyclic motifs assocated with the solvent water molecules in the structure. The ethanol group attached to the indole portion of the molecule is linked to the hydroxy O2 atom *via* hydrogen bonding interactions of the O10 solvent water. In addition, the intermolecular linkage of the molecules occurs *via* the O20 solvent water which connects the hydroxy O2 with the coordinated O10 solvent water. In addition, there is a second (Larger) cyclic motif generated by solvent water and OH groups from the hydroxymethyl and hydroxyethyl groups of the molecule. These H-bond interactions generate a two-dimensional polymeric network along the a-b plane of the structure. All O—H···O lengths and angles for these interactions are typical for hydrogen bonds as listed in Table 1.

### Experimental

The title compound was synthesized by condensation of 3-chloromethyl-5-nitrosalicylaldehyde and 9,9,9a-trimethyl-2,3,9,9a-tetrahydrooxazolo[3,2-*a*]indole in refluxing ethanol and then recrystallized from an aqueous 70% acetonitrile solution. For synthesis procedures of related compounds see Raymo & Giordani (2001).

### Refinement

H atoms present on the molecule were located in a straightforward manner using HFIX commands of *SHELXL97* with attention to hybridization of the bound atom. The H atoms from water molecules were located in a difference Fourier map. They were refined using a riding-model approximation with C—H = 0.95-0.99 Å and O-H = 0.85-0.96 Å with *U*_iso_(H)=1.2*U*_eq_(C) except methyl group and water molecule, where *U*_iso_(H)=1.5*U*_eq_(C,O).

### Crystal data

**Table:**

C_21_H_22_N_2_O_5_·2H_2_O	*Z* = 2
*M**_r_* = 418.44	*F*(000) = 444
Triclinic, *P*1	*D*_x_ = 1.330 Mg m^−^^3^*D*_m_ = 1.31 (8) Mg m^−^^3^*D*_m_ measured by picnometer
Hall symbol: -P 1	Mo *K*α radiation, λ = 0.71073 Å
*a* = 7.377 (2) Å	Cell parameters from 200 reflections
*b* = 8.868 (2) Å	θ = 1–25°
*c* = 16.817 (5) Å	µ = 0.10 mm^−^^1^
α = 94.603 (5)°	*T* = 183 K
β = 101.639 (6)°	Block, dark red
γ = 102.140 (7)°	0.10 × 0.10 × 0.10 mm
*V* = 1044.8 (5) Å^3^	

### Data collection

**Table:**

Bruker APEX CCD area-detector diffractometer	3651 independent reflections
Radiation source: fine-focus sealed tube	2472 reflections with *I* > 2σ(*I*)
graphite	*R*_int_ = 0.039
φ and ω scans	θ_max_ = 25.0°, θ_min_ = 2.4°
Absorption correction: multi-scan (*SADABS*; Sheldrick, 1999)	*h* = −8→8
*T*_min_ = 0.981, *T*_max_ = 0.990	*k* = −10→10
7525 measured reflections	*l* = −19→19

### Refinement

**Table:**

Refinement on *F*^2^	Primary atom site location: structure-invariant direct methods
Least-squares matrix: full	Secondary atom site location: difference Fourier map
*R*[*F*^2^ > 2σ(*F*^2^)] = 0.054	Hydrogen site location: inferred from neighbouring sites
*wR*(*F*^2^) = 0.134	H-atom parameters constrained
*S* = 1.02	*w* = 1/[σ^2^(*F*_o_^2^) + (0.0611*P*)^2^ + 0.0096*P*] where *P* = (*F*_o_^2^ + 2*F*_c_^2^)/3
3651 reflections	(Δ/σ)_max_ < 0.001
271 parameters	Δρ_max_ = 0.23 e Å^−^^3^
0 restraints	Δρ_min_ = −0.20 e Å^−^^3^

### Special details

**Table:**

Geometry. All esds (except the esd in the dihedral angle between two l.s. planes) are estimated using the full covariance matrix. The cell esds are taken into account individually in the estimation of esds in distances, angles and torsion angles; correlations between esds in cell parameters are only used when they are defined by crystal symmetry. An approximate (isotropic) treatment of cell esds is used for estimating esds involving l.s. planes.
Refinement. Refinement of *F*^2^ against ALL reflections. The weighted *R*-factor *wR* and goodness of fit *S* are based on *F*^2^, conventional *R*-factors *R* are based on *F*, with *F* set to zero for negative *F*^2^. The threshold expression of *F*^2^ > σ(*F*^2^) is used only for calculating *R*-factors(gt) *etc*. and is not relevant to the choice of reflections for refinement. *R*-factors based on *F*^2^ are statistically about twice as large as those based on *F*, and *R*- factors based on ALL data will be even larger.

### Fractional atomic coordinates and isotropic or equivalent isotropic displacement parameters (Å^2^)

**Table:**

	*x*	*y*	*z*	*U*_iso_*/*U*_eq_	
N1	0.4314 (3)	0.7565 (2)	0.27176 (12)	0.0266 (5)	
N2	−0.7317 (3)	0.3125 (2)	0.07265 (13)	0.0297 (5)	
O1	0.3009 (3)	0.8842 (2)	0.40555 (12)	0.0453 (5)	
H1	0.3172	0.9730	0.3903	0.054*	
O2	−0.1195 (2)	0.4576 (2)	0.33751 (11)	0.0348 (5)	
O3	−0.7048 (3)	0.3290 (2)	0.00334 (11)	0.0399 (5)	
O4	−0.8914 (3)	0.2639 (2)	0.08513 (12)	0.0439 (6)	
O5	−0.6302 (2)	0.1775 (2)	0.36144 (11)	0.0376 (5)	
H5	−0.7015	0.2285	0.3787	0.045*	
C1	0.5663 (3)	0.8684 (3)	0.24386 (15)	0.0275 (6)	
C2	0.7509 (4)	0.9396 (3)	0.28370 (17)	0.0337 (7)	
H2A	0.8074	0.9150	0.3355	0.040*	
C3	0.8489 (4)	1.0488 (3)	0.24380 (18)	0.0392 (7)	
H3	0.9755	1.1027	0.2694	0.047*	
C4	0.7670 (4)	1.0812 (3)	0.16759 (18)	0.0381 (7)	
H4	0.8380	1.1564	0.1416	0.046*	
C5	0.5822 (4)	1.0052 (3)	0.12871 (17)	0.0331 (7)	
H5A	0.5267	1.0267	0.0760	0.040*	
C6	0.4804 (3)	0.8976 (3)	0.16797 (15)	0.0251 (6)	
C7	0.2797 (3)	0.8003 (3)	0.14308 (14)	0.0248 (6)	
C8	0.2563 (4)	0.6884 (3)	0.06505 (15)	0.0308 (6)	
H8A	0.2692	0.7482	0.0192	0.046*	
H8B	0.1302	0.6172	0.0529	0.046*	
H8C	0.3545	0.6285	0.0734	0.046*	
C9	0.1368 (4)	0.9048 (3)	0.12986 (17)	0.0355 (7)	
H9A	0.1498	0.9716	0.1810	0.053*	
H9B	0.0071	0.8398	0.1132	0.053*	
H9C	0.1625	0.9697	0.0871	0.053*	
C10	0.2648 (3)	0.7159 (3)	0.21803 (15)	0.0246 (6)	
C11	0.4762 (4)	0.7097 (3)	0.35407 (15)	0.0311 (6)	
H11A	0.6030	0.6851	0.3639	0.037*	
H11B	0.3813	0.6147	0.3575	0.037*	
C12	0.4755 (4)	0.8380 (3)	0.41955 (16)	0.0392 (7)	
H12A	0.4980	0.8006	0.4738	0.047*	
H12B	0.5804	0.9288	0.4203	0.047*	
C13	0.1035 (3)	0.6165 (3)	0.23467 (15)	0.0274 (6)	
H13	0.1166	0.5811	0.2869	0.033*	
C14	−0.0685 (3)	0.5680 (3)	0.18168 (16)	0.0275 (6)	
H14	−0.0760	0.5985	0.1284	0.033*	
C15	−0.2409 (3)	0.4756 (3)	0.19654 (15)	0.0244 (6)	
C16	−0.3992 (3)	0.4350 (3)	0.13128 (15)	0.0249 (6)	
H16	−0.3883	0.4651	0.0792	0.030*	
C17	−0.5708 (3)	0.3522 (3)	0.14124 (15)	0.0249 (6)	
C18	−0.5931 (3)	0.3052 (3)	0.21741 (15)	0.0252 (6)	
H18	−0.7142	0.2517	0.2237	0.030*	
C19	−0.4402 (4)	0.3369 (3)	0.28191 (15)	0.0250 (6)	
C20	−0.2575 (4)	0.4255 (3)	0.27503 (15)	0.0266 (6)	
C21	−0.4511 (4)	0.2795 (3)	0.36328 (16)	0.0327 (7)	
H21A	−0.3487	0.2240	0.3791	0.039*	
H21B	−0.4292	0.3699	0.4054	0.039*	
O10	0.9517 (3)	0.7347 (2)	0.43717 (12)	0.0471 (6)	
H10A	1.0729	0.7979	0.4350	0.071*	
H10B	0.9319	0.6349	0.4070	0.071*	
O20	0.1635 (3)	0.3445 (3)	0.42365 (13)	0.0642 (7)	
H20B	0.1048	0.3071	0.4659	0.096*	
H20A	0.0628	0.3721	0.3872	0.096*	

### Atomic displacement parameters (Å^2^)

**Table:**

	*U*^11^	*U*^22^	*U*^33^	*U*^12^	*U*^13^	*U*^23^
N1	0.0212 (12)	0.0303 (12)	0.0277 (12)	0.0033 (10)	0.0063 (10)	0.0044 (10)
N2	0.0267 (13)	0.0250 (12)	0.0348 (14)	−0.0003 (10)	0.0046 (11)	0.0106 (10)
O1	0.0443 (13)	0.0374 (12)	0.0578 (14)	0.0064 (10)	0.0235 (11)	0.0051 (10)
O2	0.0280 (11)	0.0442 (12)	0.0284 (10)	0.0022 (9)	0.0019 (9)	0.0099 (9)
O3	0.0369 (12)	0.0498 (13)	0.0275 (11)	−0.0016 (10)	0.0049 (9)	0.0094 (9)
O4	0.0216 (11)	0.0570 (13)	0.0477 (13)	−0.0046 (10)	0.0038 (9)	0.0221 (10)
O5	0.0340 (11)	0.0422 (12)	0.0401 (11)	0.0048 (9)	0.0168 (9)	0.0148 (9)
C1	0.0214 (14)	0.0312 (15)	0.0306 (15)	0.0028 (12)	0.0119 (12)	0.0014 (12)
C2	0.0203 (15)	0.0408 (17)	0.0372 (16)	0.0033 (13)	0.0063 (12)	−0.0003 (13)
C3	0.0215 (15)	0.0390 (17)	0.054 (2)	−0.0017 (13)	0.0140 (14)	−0.0045 (15)
C4	0.0347 (17)	0.0322 (16)	0.053 (2)	0.0054 (14)	0.0255 (15)	0.0068 (14)
C5	0.0342 (17)	0.0317 (16)	0.0372 (17)	0.0073 (13)	0.0163 (14)	0.0070 (13)
C6	0.0252 (14)	0.0232 (14)	0.0272 (14)	0.0053 (11)	0.0081 (12)	−0.0006 (11)
C7	0.0238 (14)	0.0268 (14)	0.0245 (14)	0.0064 (12)	0.0065 (11)	0.0030 (11)
C8	0.0326 (16)	0.0335 (16)	0.0275 (15)	0.0082 (13)	0.0086 (12)	0.0054 (12)
C9	0.0275 (16)	0.0291 (15)	0.0486 (18)	0.0047 (13)	0.0075 (13)	0.0042 (13)
C10	0.0209 (14)	0.0281 (14)	0.0245 (14)	0.0061 (12)	0.0054 (11)	−0.0003 (11)
C11	0.0268 (15)	0.0379 (16)	0.0270 (15)	0.0074 (13)	0.0009 (12)	0.0085 (13)
C12	0.0405 (18)	0.0474 (19)	0.0272 (15)	0.0073 (15)	0.0049 (14)	0.0047 (13)
C13	0.0238 (15)	0.0321 (15)	0.0242 (14)	0.0016 (12)	0.0041 (12)	0.0066 (11)
C14	0.0284 (15)	0.0276 (14)	0.0267 (14)	0.0038 (12)	0.0090 (12)	0.0035 (11)
C15	0.0225 (14)	0.0223 (13)	0.0293 (14)	0.0054 (11)	0.0073 (12)	0.0043 (11)
C16	0.0265 (15)	0.0230 (13)	0.0256 (14)	0.0028 (11)	0.0085 (12)	0.0074 (11)
C17	0.0214 (14)	0.0227 (13)	0.0284 (14)	0.0023 (11)	0.0031 (11)	0.0041 (11)
C18	0.0223 (14)	0.0221 (13)	0.0320 (15)	0.0027 (11)	0.0090 (12)	0.0065 (11)
C19	0.0271 (15)	0.0211 (13)	0.0286 (14)	0.0056 (11)	0.0091 (12)	0.0063 (11)
C20	0.0273 (15)	0.0247 (14)	0.0269 (15)	0.0056 (12)	0.0037 (12)	0.0036 (11)
C21	0.0298 (16)	0.0367 (16)	0.0316 (15)	0.0037 (13)	0.0090 (13)	0.0085 (13)
O10	0.0411 (12)	0.0499 (13)	0.0513 (13)	0.0091 (10)	0.0144 (10)	0.0054 (10)
O20	0.0526 (15)	0.104 (2)	0.0522 (14)	0.0420 (14)	0.0186 (12)	0.0250 (14)

### Geometric parameters (Å, °)

**Table:**

N1—C10	1.331 (3)	C9—H9A	0.9797
N1—C1	1.429 (3)	C9—H9B	0.9799
N1—C11	1.471 (3)	C9—H9C	0.9803
N2—O4	1.232 (3)	C10—C13	1.416 (3)
N2—O3	1.236 (3)	C11—C12	1.520 (4)
N2—C17	1.439 (3)	C11—H11A	0.9895
O1—C12	1.414 (3)	C11—H11B	0.9895
O1—H1	0.8400	C12—H12A	0.9904
O2—C20	1.273 (3)	C12—H12B	0.9898
O5—C21	1.429 (3)	C13—C14	1.357 (3)
O5—H5	0.8405	C13—H13	0.9492
C1—C6	1.376 (3)	C14—C15	1.440 (3)
C1—C2	1.380 (3)	C14—H14	0.9503
C2—C3	1.385 (4)	C15—C16	1.393 (3)
C2—H2A	0.9507	C15—C20	1.446 (3)
C3—C4	1.382 (4)	C16—C17	1.373 (3)
C3—H3	0.9503	C16—H16	0.9504
C4—C5	1.387 (4)	C17—C18	1.408 (3)
C4—H4	0.9500	C18—C19	1.361 (3)
C5—C6	1.382 (3)	C18—H18	0.9499
C5—H5A	0.9502	C19—C20	1.442 (3)
C6—C7	1.506 (3)	C19—C21	1.509 (3)
C7—C10	1.527 (3)	C21—H21A	0.9901
C7—C8	1.538 (3)	C21—H21B	0.9898
C7—C9	1.539 (3)	O10—H10A	0.9594
C8—H8A	0.9795	O10—H10B	0.9515
C8—H8B	0.9805	O20—H20B	0.9502
C8—H8C	0.9795	O20—H20A	0.9513
			
C10—N1—C1	111.6 (2)	C13—C10—C7	128.7 (2)
C10—N1—C11	127.0 (2)	N1—C11—C12	111.2 (2)
C1—N1—C11	121.1 (2)	N1—C11—H11A	109.4
O4—N2—O3	122.4 (2)	C12—C11—H11A	109.4
O4—N2—C17	118.9 (2)	N1—C11—H11B	109.4
O3—N2—C17	118.8 (2)	C12—C11—H11B	109.4
C12—O1—H1	109.4	H11A—C11—H11B	108.0
C21—O5—H5	109.4	O1—C12—C11	111.7 (2)
C6—C1—C2	123.8 (2)	O1—C12—H12A	109.2
C6—C1—N1	108.1 (2)	C11—C12—H12A	109.3
C2—C1—N1	128.0 (2)	O1—C12—H12B	109.3
C1—C2—C3	116.1 (3)	C11—C12—H12B	109.3
C1—C2—H2A	121.9	H12A—C12—H12B	108.0
C3—C2—H2A	122.0	C14—C13—C10	125.0 (2)
C4—C3—C2	121.5 (3)	C14—C13—H13	117.5
C4—C3—H3	119.2	C10—C13—H13	117.5
C2—C3—H3	119.2	C13—C14—C15	127.7 (2)
C3—C4—C5	120.7 (3)	C13—C14—H14	116.1
C3—C4—H4	119.6	C15—C14—H14	116.2
C5—C4—H4	119.7	C16—C15—C14	117.3 (2)
C6—C5—C4	118.8 (3)	C16—C15—C20	119.2 (2)
C6—C5—H5A	120.6	C14—C15—C20	123.5 (2)
C4—C5—H5A	120.6	C17—C16—C15	120.9 (2)
C1—C6—C5	119.0 (2)	C17—C16—H16	119.5
C1—C6—C7	109.7 (2)	C15—C16—H16	119.6
C5—C6—C7	131.3 (2)	C16—C17—C18	121.4 (2)
C6—C7—C10	101.47 (19)	C16—C17—N2	119.5 (2)
C6—C7—C8	110.0 (2)	C18—C17—N2	119.1 (2)
C10—C7—C8	112.7 (2)	C19—C18—C17	119.7 (2)
C6—C7—C9	110.5 (2)	C19—C18—H18	120.2
C10—C7—C9	111.1 (2)	C17—C18—H18	120.2
C8—C7—C9	110.8 (2)	C18—C19—C20	121.1 (2)
C7—C8—H8A	109.4	C18—C19—C21	122.3 (2)
C7—C8—H8B	109.5	C20—C19—C21	116.6 (2)
H8A—C8—H8B	109.5	O2—C20—C19	119.4 (2)
C7—C8—H8C	109.5	O2—C20—C15	122.9 (2)
H8A—C8—H8C	109.5	C19—C20—C15	117.7 (2)
H8B—C8—H8C	109.5	O5—C21—C19	112.6 (2)
C7—C9—H9A	109.5	O5—C21—H21A	109.0
C7—C9—H9B	109.5	C19—C21—H21A	109.1
H9A—C9—H9B	109.4	O5—C21—H21B	109.1
C7—C9—H9C	109.5	C19—C21—H21B	109.1
H9A—C9—H9C	109.4	H21A—C21—H21B	107.8
H9B—C9—H9C	109.5	H10A—O10—H10B	110.1
N1—C10—C13	122.2 (2)	H20B—O20—H20A	102.9
N1—C10—C7	109.0 (2)		

### Hydrogen-bond geometry (Å, °)

**Table:**

*D*—H···*A*	*D*—H	H···*A*	*D*···*A*	*D*—H···*A*
O20—H20B···O10^i^	0.96	1.80	2.739 (4)	166
O10—H10B···O2^ii^	0.95	1.81	2.750 (3)	172
O20—H20A···O2	0.95	1.78	2.714 (3)	167
O10—H10A···O1^ii^	0.95	1.87	2.811 (3)	165
O5—H5···O20^iii^	0.84	1.80	2.633 (3)	175
O1—H1···O5^iv^	0.84	1.90	2.734 (3)	176

Symmetry codes: (i) −*x*+1, −*y*+1, −*z*+1; (ii) *x*+1, *y*, *z*; (iii) *x*−1, *y*, *z*; (iv) *x*+1, *y*+1, *z*.
